# Unimpressed by the Environment?—Local and Landscape Scale Effects on the Common Hamster in a Simple Agricultural Landscape

**DOI:** 10.1002/ece3.72595

**Published:** 2025-12-22

**Authors:** Pia Stein, Saskia Jerosch, Marion Pause, Christina Fischer

**Affiliations:** ^1^ Faunistics and Wildlife Conservation, Department of Agriculture, Ecotrophology, and Landscape Development Anhalt University of Applied Sciences Bernburg Germany; ^2^ Deutsche Wildtier Stiftung Hamburg Germany; ^3^ Department of Architecture, Facility Management and Geoinformation, Institute of Geoinformation and Land Surveying Anhalt University of Applied Sciences Dessau Germany

**Keywords:** agricultural intensification, common vole, endangered species, remote sensing, species protection

## Abstract

Agricultural intensification causes significant species loss in agricultural landscapes. One species particularly affected is the critically endangered common hamster ( *Cricetus cricetus* ). To counteract hamster decline, it is essential to analyze scale‐dependent factors that determine hamster occurrence and densities, especially in structurally simple landscapes. We mapped hamster burrows in the predominantly simple agricultural landscapes of Saxony‐Anhalt, Central Germany. At the local scale, we studied the effects of the hamster protection measure of high cut harvest and common vole abundance as well as satellite‐based vegetation indices. At the landscape scale, we studied the effects of landscape composition (percentage of winter cereals, oil seeds, uncultivated land, crop diversity) and configuration (edge density, mean field size, distance to the nearest forest, and urban fabric). Our results showed that hamster densities and vole abundance were negatively associated, whereas high cut harvest had a non‐significant but slightly positive effect on hamster densities. Satellite‐based vegetation indices showed no effect on hamster occurrence. At the landscape scale, the percentage of winter cereals around study field centers increased the probability of hamster occurrence, while further landscape indices had no effect, likely due to the already too simply structured landscape. Our study shows that at the local scale, attention should be paid to adapted vole pest management in order to ensure that hamsters are not further harmed. Whereas, high cut harvest as a single measure is not sufficient to stabilize hamster populations at the local scale, at the landscape scale other factors such as agri‐environmental schemes should be considered in simple landscapes to prevent the steady population decline.

## Graphical abstract

This study examined the effects of local‐ and landscape‐scale factors on the critically endangered common hamster ( *Cricetus cricetus* ) in one of Germany's largest remaining populations. At the local scale, we investigated the influence of a hamster protection measure, common vole abundance, as well as satellite‐based vegetation indices and at the landscape scale, we analyzed landscape composition and configuration. Results showed that hamster densities decreased with higher common vole abundance locally, while winter wheat increased the probability of hamster occurrence at the landscape level in spring.

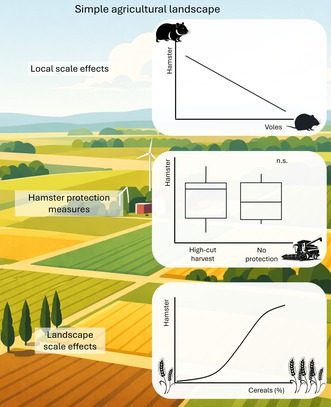

## 1 Introduction

As agricultural intensification increased in the 20th century, structurally diverse agricultural landscapes and (semi‐) natural habitats have progressively disappeared (e.g., Cousins et al. 2015; Ridding et al. 2020). As a consequence, agrobiodiversity has declined, and populations have become destabilized due to habitat fragmentation and habitat loss at a landscape scale, as well as habitat change within a short period of time (e.g., harvesting), and pesticide and fertilizer application at a local scale (Geiger et al. 2010; Gentili et al. 2014; Kleijn et al. 2009; Olivier et al. 2020; Stoate et al. 2001). These rapid changes can drive population declines at a global scale (e.g., the European Brown Hare ( *Lepus europaeus* ), Hacklander and Schai‐Braun 2019) or can lead to local extinctions (e.g., the Great Bustard ( *Otis tarda* ), BirdLife International 2023).

A prominent example of a species that has been severely affected by the intensification of agriculture is the common hamster ( *Cricetus cricetus* ) (further referred to as hamster) (Kayser et al. 2003; La Haye et al. 2014; Tissier et al. 2017, 2016). The hamster has been listed as critically endangered worldwide by the IUCN since 2019 (Banaszek et al. 2020) although it was once considered a pest species and vigorously hunted up until the 1970s (Mammen 2005). Its distribution ranges from the Asian part of Russia in the east to Belgium, France, and Germany in the west (Banaszek et al. 2020). In West and Central Europe, the hamster mainly lives on agricultural fields with deep loess or loess loamy soil (Tkadlec et al. 2012; Weidling and Stubbe 1998a). Since the 1970s the hamster has lost about 74% of its Central and East European distribution area and might go extinct within the next two decades (Surov et al. 2016). Only in Romania and the Czech Republic was the conservation status of the hamster considered favorable between 2013 and 2018 (European Environment Agency, n.d.).

To prevent further population decline, the hamster is legally protected by the EU habitat directive (Annex IV: animal and plant species of community interest in need of strict protection, exception Hungary, listed in Annex V: harvest of the species is allowed) and the Bern Convention (Appendix II: strictly protected fauna species). There are a variety of protection measures; for example plowing late in October and not deeper than 25 cm as well as smaller field sizes to save and increase the survival rate of the hamster (Weinhold 2009). Furthermore, hamsters can be supported by hamster survival stripes (La Haye 2007), wildflower stripes or fields (Deutsche Wildtierstiftung 2022; Fischer and Wagner 2016), hamster‐friendly crops such as alfalfa in the crop rotation or high cut harvest (Deutsche Wildtierstiftung 2022), which secure vegetation cover and food resources over the whole vegetation period (Weinhold 2009). High cut harvest seems to be one of the simpler protection measures to be implemented as it can be done while harvesting cereals in July or August. This method involves harvesting a 12 m strip of cereal just below the ear as well as leaving the rest of the field with at least 30 cm of stubble and is supposed to provide better cover from predators after harvest (Deutsche Wildtierstiftung 2022).

Another measure to prevent the hamster from getting harmed is the legal restriction of rodenticides in regions with hamster occurrences (e.g., Germany: Bundesamt für Verbraucherschutz und Lebensmittelsicherheit 2019; France: Ministre de l'agriculture et de la souveraineté alimentaire 2024). However, this can result in conflicts in regions where the population pressure of pest species such as the common vole ( *Microtus arvalis* ) can cause massive crop damage and reduce harvests (Jacob et al. 2014). Hamsters and common voles have similar habitat preferences in agricultural landscapes; for example, voles also prefer higher vegetation cover and density after harvest (Banaszek et al. 2020; Fischer and Schröder 2014; Jacob et al. 2014). However, little is known about the possibility and mechanisms of their co‐occurrence.

On a landscape scale, Fischer and Wagner (2016) found that at least 60% of arable land around wildflower fields increased the probability of occurrence of the hamster to more than 50% and that a closer distance to forests had a negative effect while settlements were found to have no effect. Hamsters prefer cereal fields like winter wheat, perennial clover, or alfalfa fields for the establishment of their burrows (Albert et al. 2011). However, the hamster is especially affected by crop harvest, as cover and food resources become lost within a few days (La Haye et al. 2010; Out et al. 2011). In simple agricultural landscapes with large fields, the issue is even more pronounced and a major driver for the hamsters' endangerment (Kayser and Stubbe 2003). Furthermore, large fields with monocultures, which are related to low crop diversity and low edge density, do not provide hamsters with enough resources to ensure their survival and reproductive success (Tissier et al. 2017, 2021). Whereas studies on local and landscape scale effects on hamster occurrence have been conducted in complex landscapes such as Bavaria and Hesse, Germany (Albert et al. 2011; Fischer and Wagner 2016) or in peri‐urban or urban areas (Feoktistova et al. 2020; Katzman et al. 2023), structurally simple landscapes with a high percentage of arable land and large agricultural fields have rarely been studied (Kayser et al. 2003).

To study the impact of local and landscape scale effects as well as the co‐occurrence between common voles and hamsters in a structurally simple agricultural landscape, we observed the density of hamster summer burrows and the occurrence of reopened winter burrows in Saxony‐Anhalt, Central Germany. The study area is a simple agricultural landscape and hosts one of the largest remaining hamster populations in Germany (Meinig et al. 2020). As local scale parameters, we examined the impact of the hamster protection measure high cut harvest and common vole abundance on hamster density in summer. Furthermore, we analyzed the effects of vegetation cover and crop residue using broadband multispectral indices calculated from Copernicus Sentinel‐2 MSI L2A data on the occurrence of reopened winter burrows. At the landscape scale, we analyzed the occurrence of reopened winter burrows in regard to the surrounding landscape composition and configuration in terms of percentage cover of different crop types such as winter cereals and crop diversity as well as the distance to the nearest urban fabric or forest to answer the following research questions:

1. How effective is the hamster protection measure, high cut harvest, at a local scale for hamster densities?
2. Does common vole abundance impact the hamster density and do vegetation cover and crop residue cover affect hamster occurrence at a local scale?
3. Which landscape scale effects impact the hamster occurrence in a structurally simple agricultural landscape?

## 2 Methods

### 2.1 Study Area

**FIGURE 1:**
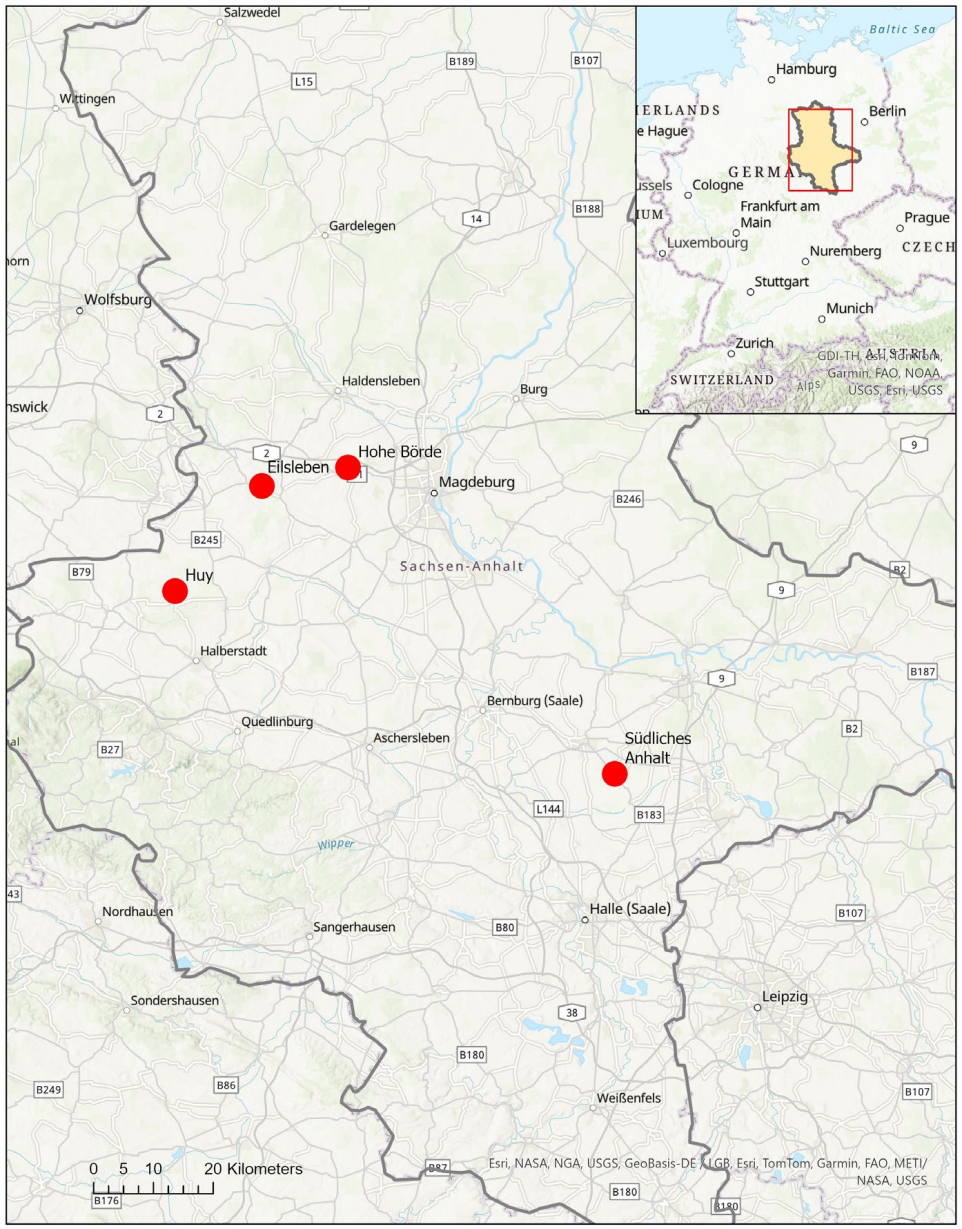
The four selected study regions in Saxony‐Anhalt with known hamster occurrences (red dots), view of the federal state Saxony‐Anhalt, source: GeoBasis‐DE/BKG 2023 (Data adjusted); background map: World Topographic Map and map source: Esri, CGIAR, USGS, GeoBasis‐DE/LGB, TomTom, Garmin, Foursquare, FAO, METI/NASA, GDI‐TH, NOAA.

The study was conducted from August 2022 until September 2023 in Saxony‐Anhalt in Central Germany. We surveyed four different regions with known hamster occurrences. The two regions Eilsleben and Hohe Börde were situated in the Magdeburger Börde, Huy in the northern Harz foreland and Südliches Anhalt in the eastern part of the Saalekreis district (Figure 1). The size for the regions was set at a maximum of 5000 ha. The minimum distance between regions was 8 km and the maximum distance was 71 km. All regions are characterized by fertile black loess soil. The mean annual rainfall is between 500 and 600 mm (years 1991–2020 averaged) (Landesanstalt für Landwirtschaft und Gartenbau Sachsen‐Anhalt 2024b). The landscape is structurally simple and characterized by on average 79% arable land which was dominated by winter wheat as the most abundant crop and the mean field size was 11 ha. However, fields with the same crops often appeared next to each other without any significant boundary. Agricultural data were accessed from the database of the “Integriertes Verwaltungs‐ und Kontrollsystem” (InVeKoS) of 2022 and 2023.

#### 2.1.1 Field Site Selection for Summer Burrow Assessment

To study the effectiveness of the hamster protection measure high cut harvest on the hamster density in fall 2022 and 2023 (hereafter referred to as summer burrows), we selected 10 study fields with high cut harvest (Figure A.1) and 6 study fields with conventional harvest (Table 1). The mean field size was 15.75 ± 2.75 ha (min. = 2.93 ha, max. = 39.84 ha). Most of the study fields were cultivated with winter cereals (*n* = 14), whereas summer cereals as well as agroforestry with legumes and winter cereals made up one field each. Field centers within each region had a mean distance of 412.9 ± 65.5 m (min. = 92.1 m, max. = 959.1 m).

**TABLE 1:** Sampling effort (*n*) per region to study local scale effects in terms of hamster protection measure, conventional harvest and common vole abundance as well as satellite‐based vegetation indices and landscape scale effects on hamster densities or occurrence. The effects of hamster protection measure versus conventional harvest and common vole abundance were tested for summer burrows, while vegetation indices and landscape scale effects were tested for reopened winter burrows.

Parameter	Huy			Eilsleben			Hohe Börde			Südliches Anhalt			Sum
	Fall 2022	Spring 2023	Fall 2023	Fall 2022	Spring 2023	Fall 2023	Fall 2022	Spring 2023	Fall 2023	Fall 2022	Spring 2023	Fall 2023	
Hamster protection measure	—	—	—	3	—	—	2	—	—	2	—	3	10
Conventional harvest	2	—	—	2	—	—	1	—	—	—	—	1	6
Common vole abundance	—	—	—	4	—	—	3	—	—	6	—	—	13
Vegetation indices/landscape scale effects	—	3	—	—	6	—	—	5	—	—	5	—	19

#### 2.1.2 Field Site Selection for Reopened Winter Burrow Assessment

To account for landscape scale effects on reopened winter burrows, additional fields were selected to cover a landscape complexity gradient. In total, 19 fields, of which 15 were added, were selected within the same four study regions mentioned before. Crops on the fields were winter cereals (*n* = 10), summer cereals (*n* = 3), legumes (*n* = 3), and fields prepared for or freshly planted with sugar beet and maize (*n* = 3). Fields were selected to be distributed across each study region and field centers had a mean distance of 1272.6 ± 145.4 m (min. = 750.3 m, max. = 3080.7 m) per region.

### 2.2 Mapping of Hamster Burrows

We mapped summer burrows of hamsters in August and September 2022 and 2023 respectively after the crops were harvested, as they can indicate population dynamics of hamsters over the summer (Weidling and Stubbe 1998b). Reopened winter burrows were mapped in late April and May 2023, as they can be a good measure for population size estimations of animals that successfully hibernate, as they are usually used by individual animals (Weidling and Stubbe 1998b). Burrows were mapped by systematically searching each field by several observers in neighboring transects. The distance between transects was between 2 and 5 m (Weidling and Stubbe 1998b) and depended on the visibility of hamster burrows in relation to crop cover and height. Burrow entrances of adult hamsters are usually 6 to 9 cm wide with a minimum depth of 30 cm (Eisentraut 1928; Grulich 1981; Weinhold and Kayser 2006). Summer burrows often consist of multiple entrances and escape tunnels while the reopened winter burrows usually consist of one deep escape tunnel. The locations of hamster burrows were mapped using mobile GPS devices; if one burrow consisted of multiple entrances a central point was taken. We mapped 10 ha per field, or the entire field if smaller than 10 ha. To calculate the hamster density, the number of hamster burrows per field was rescaled to hamster burrows per ha.

### 2.3 Assessment of Common Vole Abundances

Common vole abundance was mainly assessed on the same fields where hamsters were documented by mapping burrows in fall 2022 before the soil was plowed. We additionally sampled four summer cereal fields that could not be taken into account for the protection measure analysis and excluded three previously sampled fields as the soil was broken up before the common vole mapping (*n* = 13, mean field size: 8.97 ± 1.94 ha, Table 1). In spring 2023 we did not map vole burrows as we rarely observed vole activities in the fields and it was logistically not possible to sample the vole burrows after harvest in 2023. Following Esther et al. (2014), four 16 × 16 m squares were sampled at fields larger than 2.5 ha. For smaller fields, one 16 × 16 m square was sampled. For each square, all burrow entrances were systematically searched, marked, and closed with a layer of soil. The next day the closed points were checked for reopening and an active vole burrow index was calculated (Esther et al. 2014). Then, the active vole burrow index per ha was calculated (hereafter referred to as common vole abundance).

### 2.4 Satellite‐Based Vegetation Cover Estimation

We examined the effect of green vegetation cover and crop residue cover calculated for 24 August 2022 as the beginning of hibernation on the occurrence of reopened winter burrows. Therefore, we used two broadband multi‐spectral indices extracted from atmospherically corrected Copernicus Sentinel‐2 MSI L2A data (Copernicus Sentinel‐2 [Processed by ESA] 2022) under clear sky conditions. We calculated the Normalized Difference Vegetation Index (NDVI) (see Equation 1) to estimate green vegetation cover of our study fields as well as Normalized Difference Tillage Index (NDTI) (see Equation 2) as an estimate of crop residue on fields under dry conditions (Cai et al. 2018; Quemada and Daughtry 2016; Yue and Tian 2020).

(1) $$\text{NDVI}=\frac{\left(\mathrm{NIR}-\mathrm{RED}\right)}{\left(\mathrm{NIR}+\mathrm{RED}\right)}$$

(2) $$\text{NDTI}=\frac{\left(\text{SWIR}1-\text{SWIR}2\right)}{\left(\text{SWIR}1+\text{SWIR}2\right)}$$

For the NDVI calculation, band 4 was used for RED (reflection value within the red spectra) and band 8 was used for NIR (reflection value within the near‐infrared spectra). For the NDTI calculation, bands 11 and 12 were used for SWIR1 and SWIR 2 (representing reflection values for two positions within the short‐wave infrared) (Yue and Tian 2020). After calculating NDVI (10 m resolution) and NDTI (20 m resolution), the mean value of each study field was computed (Table 2) and used for statistical analysis.

**TABLE 2:** Overview of the analyzed local (common vole abundance: *n* = 13, vegetation indices: *n* = 19) and landscape parameters (*n* = 19) to study the effects on the density of hamster summer burrows and occurrence of reopened winter burrows. Crop type cover and landscape metrics were calculated within a radius of 500 m around the study field center. The definitions of the landscape metrics (crop diversity, mean field size, edge density) are derived from the landscape metrics package from Hesselbarth et al. (2019).

Variable	Mean ± SE	Minimum	Maximum	Definition
(a) Local parameters				
Common vole abundance: Active vole burrow index	583.85 ± 99.09	30	1090	Number of reopened vole burrows per ha, 24 h after closing them
Normalized Difference Vegetation Index (NDVI)	0.19 ± 0.02	0.1	0.51	See Equation (1), estimates vegetation cover, the mean of each study field was calculated
Normalized Difference Tillage Index (NDTI)	0.09 ± 0.01	0.03	0.16	See Equation (2), estimates crop residue and tillage practice, the mean of each study field was calculated
(b) Landscape composition and configuration				
Winter cereals (%)	45.41 ± 3.99	5.74	78.17	Cover of winter cereals such as winter wheat
Oil seeds (%)	10.92 ± 2.21	0.00	30.79	Cover of oil seeds like oilseed rape, sunflower
Uncultivated land (%)	26.44 ± 4.94	2.55	89.39	Cover of uncultivated fields in fall (fields, where crops like maize, root crops, summer cereals, legumes were sown in spring)
Crop diversity	1.51 ± 0.05	1.15	1.91	Describes the crop diversity calculated using the Shannon diversity index
Edge density (m/ha)	134.14 ± 8.35	86.2	209.22	All edges in relation to the landscape area
Mean field size (ha)	4.19 ± 0.51	0.8	8.36	Mean field sizes within the landscape area
Distance to the nearest forest (m)	493.44 ± 85.35	39.90	1336.50	Euclidean distance from the study field to the nearest forest
Distance to the nearest urban fabric (m)	418.31 ± 87.74	0.00	1265.90	Euclidean distance from the study field to the nearest urban fabric

### 2.5 Landscape Scale Parameters

To test the effects of landscape composition and configuration on the occurrence of reopened winter burrows of 2023, parameters were calculated for a 500 m radius around the center of each study field. The radius was chosen based on the hamsters' home range size of 1.85 to 3 ha (males) and 0.22 to 0.35 ha (females) and moving distances from usually less than 500 m (Kayser and Stubbe 2003; Kupfernagel 2007; van Wijk et al. 2011). For landscape composition, the percentage cover of functional crop types was calculated and crop types with a maximum of 30% and more were chosen (winter cereals, oil seeds, uncultivated land, Table 2). Crop diversity was calculated using the Shannon diversity index for all occurring crops to include the effects of rare crops. We did not include flower stripes or other agri‐environmental schemes as they did not appear in the 500 m radius. For landscape configuration, we calculated edge density (in m per ha), mean field size (in ha), as well as Euclidean distance to the nearest urban fabric and forest (in m, Table 2). For calculations of landscape parameters, the R package landscapemetrics (Hesselbarth et al. 2019) in R Version 4.3.3 (R Core Team 2024) was used. Euclidian distances were measured using ArcGIS Pro 3.0.3.

### 2.6 Statistical Analysis

For all statistical analysis we used the program R Version 4.3.3 (R Core Team 2024). The density of hamster summer burrows per ha in relation to protection measure (high cut harvest vs. no protection) as well as the common vole abundance were analyzed using linear mixed‐effects models (LME) with the nlme package (Pinheiro et al. 2023). Spatial autocorrelation was considered by setting the study region (*n* = 4) as a random effect. To account for normality and heteroscedasticity the variable summer burrows per ha was square root‐transformed for the effects of the protection measure and log (*y*)‐transformed to study the effects of common vole abundance. For both models residual diagnostics indicated that the transformation improved the distribution of residuals in the QQ‐plot and showed no clear pattern in the residual versus fitted plot, suggesting homoscedasticity.

To test the local effect of green vegetation (NDVI) and crop residue (NDTI) as well as effects of landscape composition (percentage of winter cereals, oil seeds, uncultivated land, and crop diversity) and configuration (edge density, mean field size, distance to the nearest forest) on the presence or absence of reopened winter burrows we used generalized linear models (GLM) for binary data (family = binominal, link = logit) respectively. Previously, landscape scale parameters (Table 2) have been checked for collinearity by using Spearman rank correlation and setting the threshold at *r*_s_ < 0.7 (Dormann et al. 2013). Only non‐multicollinear variables have been used for analysis (distance to the nearest urban fabric was excluded; Table A.1). Because of the small sample size, we modeled the effects of local scale parameters as well as landscape composition and configuration separately. In each case the best model was chosen with the corrected AIC (AICc) for small sample sizes of the possible models using the R package MuMIn (Bartoń 2023). We visualized the regression lines of the best models using visreg (Breheny and Burchett 2017). In our results, we show mean values and their standard errors.

## 3 Results

We found 19.13 ± 7.54 summer burrows per ha in 2022 (*n* = 16) and 2.15 ± 0.53 summer burrows per ha in 2023 (*n* = 4). Furthermore, we found 0.84 ± 0.61 reopened winter burrows per ha in 2023 (*n* = 19).

### 3.1 Local Scale Effects

**FIGURE 2:**
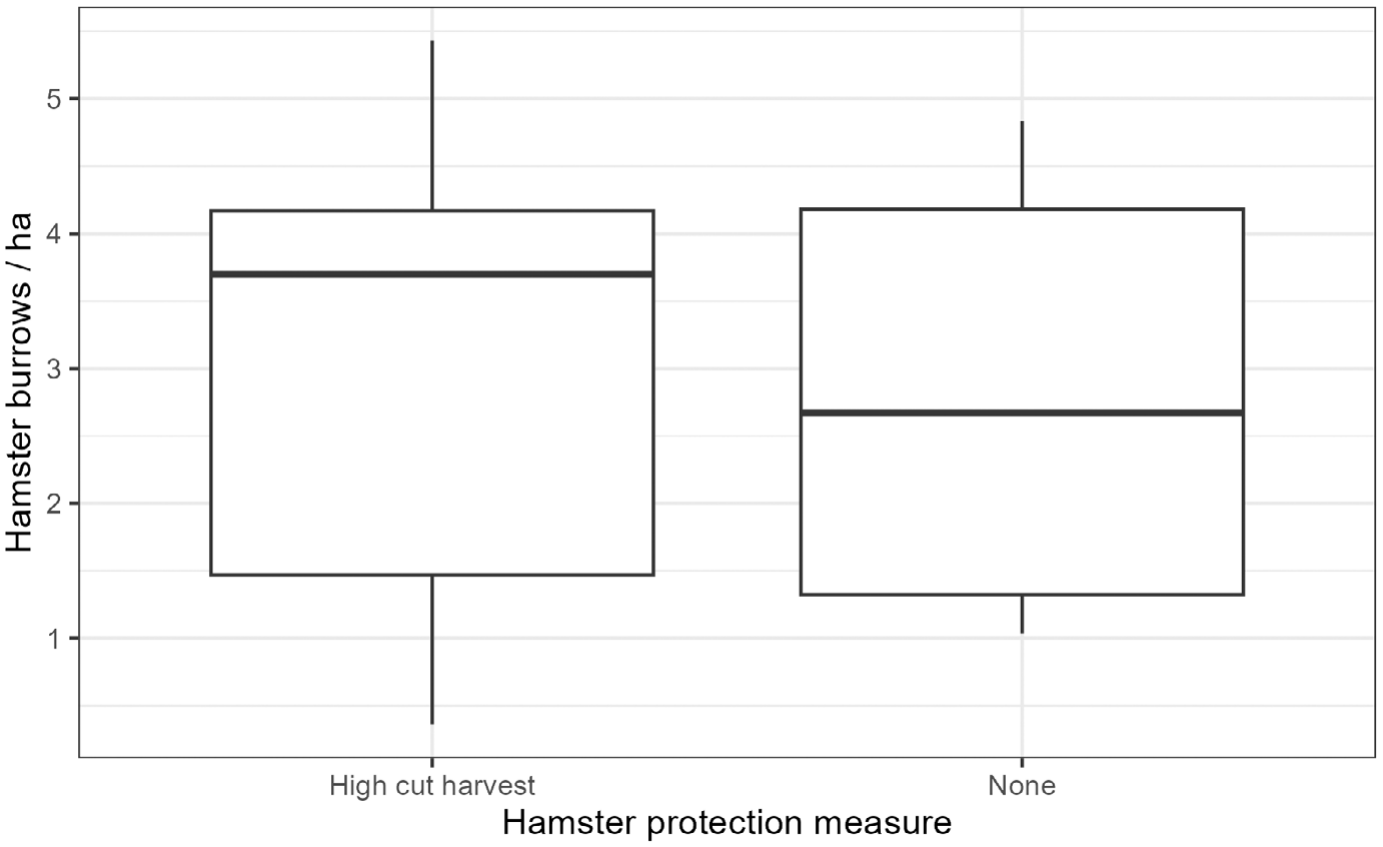
Comparison of hamster summer burrows per ha between fields with the hamster protection measure high cut harvest (left) and without a protection measure (None, right).

There was no significant difference in the density of hamster summer burrows per ha between fields with high cut harvest (3.0 ± 0.6 burrows per ha) compared to fields without any protection measures (2.7 ± 0.6 burrows per ha; Estimate = −0.04 ± 0.31, *t*_11_ = −0.12, *p*‐value = 0.91; Figure 2).

**FIGURE 3:**
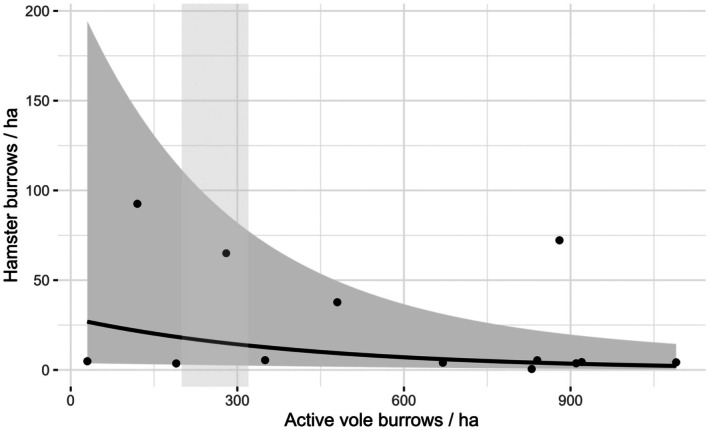
Hamster summer burrows per ha in relation to common vole abundance per ha calculated form the active vole burrow index for fall 2022. The light gray area marks the threshold of 200 to 320 active vole burrows for the allowed use of rodenticides (Landesanstalt für Landwirtschaft und Gartenbau Sachsen‐Anhalt 2024a). The gray area represents the confidence band. Raw data are shown with black points.

On average, common vole abundance was 583.85 ± 99.09 active vole burrows per ha. Our results show that an increase in the common vole abundance was associated with a decrease of the density of hamster summer burrows (Estimate: −0.002 ± 0.001, *t*_9_ = −2.46, *p* = 0.04; Figure 3).

In the best model fit both satellite‐based vegetation indices, green vegetation cover estimated by NDVI and crop residue cover estimated by NDTI, were excluded (Table A.2) and thus did not impact the occurrence of reopened winter burrows.

### 3.2 Impacts of Landscape Scale Parameters

**FIGURE 4:**
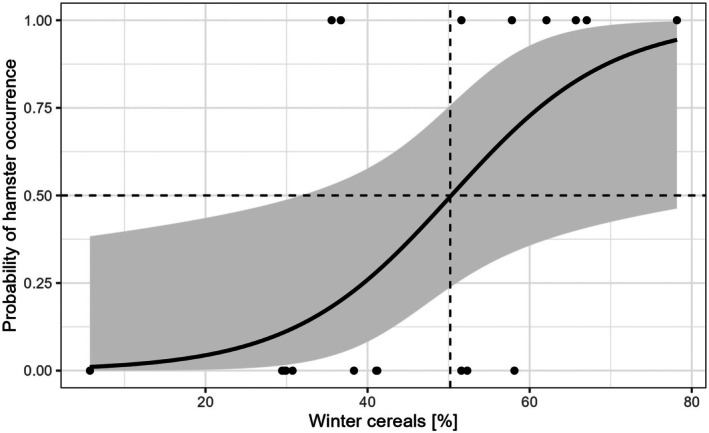
Probability of the occurrence of reopened hamster winter burrows with increasing cover of winter cereals in % within a 500 m radius around study field centers. Dashed lines represent a threshold level of 50% occurrence probability. The gray area represents the confidence band. Raw data are shown with black points.

The best model fit for landscape composition only included the cover of winter cereals (Table 3, Table A.3), which increased the probability of occurrence of reopened winter burrows. Thereby, the probability of hamster occurrence was higher than 50% at a coverage of at least 50% winter cereals within a radius of 500 m around study field centers (Figure 4). For landscape configuration, only edge density remained in the best model but had no significant effect on the occurrence of reopened winter burrows (Table 3, Table A.4).

**TABLE 3:** Summary of the best general linear model to analyze the landscape scale effects (composition and configuration respectively) on the occurrence of hamster winter borrows. Shown are the parameter estimates, standard error (SE), the *z*‐value and *p*‐value. Parameters indicated by “—” did not appear in the best model.

Parameter	Estimate	SE	*z*‐value	*p*
Landscape composition				
(Intercept)	−5.11	2.33	−2.2	0.03
Winter cereals	0.1	0.05	2.17	0.03
Oil seeds	—	—	—	—
Uncultivated land	—	—	—	—
Crop diversity	—	—	—	—
Landscape configuration				
(Intercept)	3.22	2.17	1.48	0.14
Edge density	−0.03	0.02	−1.63	0.10
Mean field size	—	—	—	—
Distance to nearest forest	—	—	—	—

## 4 Discussion

### 4.1 Local Scale Effects on Hamster Densities

We did not find an impact of high cut harvest on the density of summer burrows compared to fields without protection measures. However, we cannot exclude or validate a positive effect of high cut harvest on reproductive success or survival during hibernation, as we did not study the same fields during the three sampling periods. Although high cut harvest increases the cover of taller cereal stubbles and thus possibly offers hamsters protection from predators, such conservation measure cannot effectively counter the lack of diverse food resources that seem to affect the hamster after harvest (La Haye et al. 2010; Out et al. 2011). However, high cut harvest could provide sufficient cover and time for relocation of hamsters to other fields or flower stripes with better habitat attributes and nutrition. While Kourkgy et al. (2019) found that 71% of their marked hamsters remained within a harvested field; a certain percentage of hamsters still disperse to fields with potentially better habitat conditions, highlighting the importance of effective local protection measures. Other local measures such as the sowing of hamster‐friendly crops like alfalfa and clover (Weinhold 2009), the diversification of crops (Tissier et al. 2021), as well as hamster survival stripes (La Haye 2007), or flower stripes (Fischer and Wagner 2016) can supplement high cut harvest in structurally simple landscapes such as our study regions or may be more suitable for hamster conservation.

Looking at inter‐specific interactions at a local scale, common voles and hamsters are synanthropic species in European agricultural landscapes and can co‐occur (Banaszek et al. 2020; Jacob et al. 2014). Here we could show that there is a negative association between common vole abundance and hamster summer burrows especially emphasized when vole abundances are high. This negative association of common voles and hamsters could indicate competition for resources between these two species at high common vole abundances. Thereby, common voles might be the more adapted competitor due to higher reproduction rates and high abundances (Jacob et al. 2014) even though they can occasionally become prey to the hamster (Tissier et al. 2019). Common vole abundance often increases in the months after harvest (Cornulier et al. 2013; Heroldová et al. 2021), which is a critical time for hamster survival (La Haye et al. 2014; Out et al. 2011). Especially food competition in vole outbreak years may affect the hamsters before hibernation which can be added to other impacts like predation pressure and weather (Łopucki et al. 2024; Villemey et al. 2013), highlighting the need for diverse, high‐quality food resources, especially in simple agricultural landscapes (Tissier et al. 2017, 2019). A further explanation for the negative association between common voles and hamsters could be the higher reproduction rates of common voles after harvest and their higher resilience to modern agricultural practices that are considered harmful for the common hamster (Jacob et al. 2014; Janova et al. 2011; La Haye et al. 2014). Further, our results may be affected by the fact that common voles may benefit more from high cut harvest due to the higher vegetation cover (Fischer and Schröder 2014) than hamsters (Fischer and Wagner 2016) especially after harvest.

In Germany, control measures of common voles can be implemented in agricultural fields from more than 200 to 320 active vole burrows/ha (Landesanstalt für Landwirtschaft und Gartenbau Sachsen‐Anhalt 2024a). This creates a conflict of interest between farmers and hamster conservation, as it is prohibited to use rodenticides in areas where hamsters occur (Bundesamt für Verbraucherschutz und Lebensmittelsicherheit 2019). With our results, we can underline the importance of the prohibition of rodenticides in areas with potential hamster occurrence, as both species co‐occur even at high vole densities. There was approximately a reduction from 27 to 13 hamster burrows per ha with the increase to the economic threshold level of up to 320 vole burrows per ha. As hamster densities are already so low, that a local and also global extinction of the species is likely within the next 20 years (Surov et al. 2016), a further weakening of populations by rodenticides, even at low hamster densities, could further exacerbate this trend. Furthermore, against this background, possible methodological limitations such as missed burrows due to vegetation cover, observer bias (Weidling and Stubbe 1998b), and effects of vole population cycles (Cornulier et al. 2013) must also be carefully considered.

In our results green vegetation cover and crop residue estimated by satellite‐based vegetation indices were not related to the occurrence of reopened winter burrows at the local scale. This contradicts other studies that found that vegetation cover is important for hamster survival after harvest (Out et al. 2011). However, the used satellite‐based indices do not fully capture the small‐scale variability on the study fields and might not be the best method to assess the variability in spatial patterns on the fields. Despite this limitation, our results indicate that our study fields, especially with winter cereals, lie fallow after harvest. In simple agricultural landscapes, this likely leads to further homogenization at the local field scale, resulting in low cover of green vegetation (0.19 ± 0.02) compared to other habitats (e.g., NDVI of forests and golf course grass ≈0.8, see Huang et al. 2021) while the fields are mainly covered by crop residue (0.09 ± 0.01) (Cai et al. 2018).

### 4.2 Landscape Scale Effects on Hamster Occurrence

Our study showed that the probability of finding reopened winter burrows increased with an increasing amount of winter cereals at the landscape scale. Thereby there was a 50% probability of finding reopened winter burrows at a threshold of 50% winter cereals within a 500 m radius around study field centers. This aligns with hamsters' habitat preference for winter cereals specifically winter wheat (Albert et al. 2011; Bald et al. 2021; Kayser and Stubbe 2003; Kourkgy et al. 2019). In Saxony‐Anhalt winter wheat makes up about a quarter of the arable land (25.87% ± 0.62% average from InVeKos—data 2017 to 2023 for all of Saxony‐Anhalt), yet the population decline continues (Meinig et al. 2020). This might be related to malnutrition caused by monocultures, a reduction in the reproductive period, and a reduced probability of survival. For example, Bald et al. (2021) showed that hamster abundance in harvested cereal fields declined while it increased in carrot fields indicating that special crops might contribute to stabilizing hamster populations. Similarly, Tissier et al. (2021) suggest wheat‐soy, maize‐sunflower or radish associations (e.g., by intersowing). While we could not examine the effects of flower stripes on hamster occurrence due to the lack of presence, Fischer and Wagner (2016) found that in comparison to arable fields hamsters prefer perennial flower stripes for hibernating. Hence, diversifying simple agricultural landscapes could provide enough resources for the hamster and thus improve hamster conservation.

However, we did not find an impact of landscape composition in terms of crop diversity and landscape configuration regarding edge density, mean field size, and distance to the nearest forest. The lack of effect of crop diversity might be due to the inclusion of hamster‐unfriendly crops such as oilseed rape (Albert et al. 2011), which increases the crop diversity but without any positive ecological effect for the hamster (Li and Wu 2004). Additionally, in this study, no valuable (micro)habitats such as field margins or flower stripes contributed to the habitat diversity as they did not occur in a 500 m radius around study field centers. Our results did not indicate a positive effect of increasing distance to forests on hamster occurrence as observed in the more complex agricultural system in Bavaria (Fischer and Wagner 2016) as forests made up a small proportion in our study areas. Given that our study area is already simplified to such an extent, with large fields and low edge density, landscape scale effects are likely to be of limited relevance for hamster occurrence making local in‐crop management more important. In more complex landscapes and regions with more variable topography, results may be further influenced by factors such as altitude, slope or rivers (Tkadlec et al. 2012).

## 5 Conclusion

In our simple agricultural landscape of Saxony‐Anhalt, Central Germany the hamster protection measure high cut harvest did not enhance the density of hamster summer burrows significantly compared to fields without measures at the local scale as has been shown in other small mammal–agricultural systems (Ruscoe et al. 2023). However, such measures in combination with additional protection measures that ensure food resources and further cover can increase hamster populations (Tissier et al. 2019). Moreover, the density of summer burrows was negatively associated with common vole abundance making it important to implement an adapted vole management that does not harm the hamster. Further research on the co‐occurrence of these two species over multiple years is needed to include common vole cycles and outbreaks (Cornulier et al. 2013). While we could not find effects of green vegetation and crop residue for the analyzed Sentinel‐2 data on hamster occurrence, further remote sensing methods and spatial high‐resolution observations (e.g., by drones) have the potential to characterize hamster habitats and their spatial and temporal dynamics, as similar research on other species is already available (Marston et al. 2023; Singh et al. 2017; Thürkow et al. 2025) and should be included in future studies. At the landscape scale, our results show that winter cereals had a positive effect on the occurrence of reopened winter burrows. However, we did not find further landscape scale effects of crop diversity, mean field size, or edge density for hamster occurrence, likely due to the already highly simplified landscape. We could not account for the effects of hamster‐friendly crops, other than winter cereals, or other protection measures (Fischer and Wagner 2016; Kayser and Stubbe 2003; Out et al. 2011) since these did not occure in our study area. Considering the need for more diverse landscapes and possible conservation measures to be implemented at the local scale, the hamster can become an umbrella species, especially in largely simple agricultural landscapes, whose conservation can benefit other species, such as birds, some carnivores or ground‐dwelling invertebrates (reviewed in Hędrzak et al. 2021; Kletty et al. 2019).

## Author Contributions

**Pia Stein:** data curation (equal), formal analysis (equal), investigation (equal), methodology (equal), visualization (equal), writing – original draft (equal), writing – review and editing (equal). **Saskia Jerosch:** data curation (equal), investigation (equal), resources (equal), writing – review and editing (equal). **Marion Pause:** funding acquisition (equal), writing – review and editing (equal). **Christina Fischer:** conceptualization (equal), funding acquisition (equal), methodology (equal), project administration (equal), supervision (equal), writing – review and editing (equal).

## Funding

The project was funded by the Ministerium für Wissenschaft, Energie, Klimaschutz und Umwelt des Landes Sachsen‐Anhalt (project number: U05/2022) and by the Bundesministerium für Landwirtschaft, Ernährung und Heimat (project number: 28DE205A21) managed by the Federal Agency for Agriculture and Food.

## Conflicts of Interest

The authors declare no conflicts of interest.

## Supporting information


**Data S1:** ece372595‐sup‐0001‐DataS1.xlsx.

**Appendix S1:** ece372595‐sup‐0002‐AppendixS1.docx.

## Data Availability

The data generated and used for the analysis during this study is provided in the Supporting Information. Please be aware that we cannot publish the coordinates of our fields and have therefore anonymized the field locations.

## References

[ece372595-bib-0001] Albert, M. , T. E. Reiners , and J. A. Encarnação . 2011. “Distribution of Common Hamsters ( *Cricetus cricetus* ) in Relation to Landscape Scale Crop Composition in Hesse (Central Germany).” 18th Meeting of the International Hamster Workgroup, 2011, Strasbourg, France.

[ece372595-bib-0002] Bald, V. , F. A. Boetzl , and J. Krauss . 2021. “Where Do Hamsters Go After Cereal Harvest? A Case Study.” Basic and Applied Ecology 54: 98–107. 10.1016/j.baae.2021.04.008.

[ece372595-bib-0003] Banaszek, A. , P. L. Bogomolov , N. Feoktistova , et al. 2020. “*Cricetus cricetus*.” The IUCN Red List of Threatened Species 2020: e.T5529A111875852. 10.2305/IUCN.UK.2020-2.RLTS.T5529A111875852.en.

[ece372595-bib-0004] Bartoń, K. 2023. “MuMIn: Multi‐Model Inference, R Package Version 1.47.5.” 10.32614/CRAN.package.MuMIn.

[ece372595-bib-0005] BirdLife International . 2023. “ *Otis tarda* .” The IUCN Red List of Threatened Species 2023: e.T22691900A226280431. 10.2305/IUCN.UK.2023-1.RLTS.T22691900A226280431.en.

[ece372595-bib-0006] Breheny, P. , and W. Burchett . 2017. “Visualization of Regression Models Using Visreg.” R Journal 9: 56–71. 10.32614/RJ-2017-046.

[ece372595-bib-0007] Bundesamt für Verbraucherschutz und Lebensmittelsicherheit . 2019. “BVL erläutert Änderungen bei den Anwendungsbestimmungen für die Pflanzenschutzmittel ‘Ratron Gift‐Linsen’, ‘Ratron Gift‐Linsen Forst’, ‘Ratron Giftweizen’, ‘Ratron Schermaus‐Sticks’ und ‘ARVALIN’.” https://www.bvl.bund.de/SharedDocs/Fachmeldungen/04_pflanzenschutzmittel/2019/2019_11_07_Fa_Anwendungsbestimmungen_Rodentizide.html.

[ece372595-bib-0008] Cai, W. , S. Zhao , Z. Zhang , F. Peng , and J. Xu . 2018. Comparison of Different Crop Residue Indices for Estimating Crop Residue Cover Using Field Observation Data. 7th International Conference on Agro‐Geoinformatics, 2018, Hangzhou, China, 1–4. 10.1109/Agro-Geoinformatics.2018.8476112.

[ece372595-bib-0009] Copernicus Sentinel‐2 (Processed by ESA) . 2022. Sentinel‐2 MSI Level‐2A BOA Reflectance Product. Collection 1. European Space Agency. 10.5270/S2_-znk9xsj.

[ece372595-bib-0010] Cornulier, T. , N. G. Yoccoz , V. Bretagnolle , et al. 2013. “Europe‐Wide Dampening of Population Cycles in Keystone Herbivores.” Science 340: 63–66. 10.1126/science.1228992. 23559246

[ece372595-bib-0011] Cousins, S. A. O. , A. G. Auffret , J. Lindgren , and L. Tränk . 2015. “Regional‐Scale Land‐Cover Change During the 20th Century and Its Consequences for Biodiversity.” Ambio 44: 17–27. 10.1007/s13280-014-0585-9. PMC4288995 25576277

[ece372595-bib-0012] Deutsche Wildtierstiftung . 2022. Leitlinien Feldhamsterschutz. Self‐Publishing.

[ece372595-bib-0013] Dormann, C. F. , J. Elith , S. Bacher , et al. 2013. “Collinearity: A Review of Methods to Deal With It and a Simulation Study Evaluating Their Performance.” Ecography 36: 27–46. 10.1111/j.1600-0587.2012.07348.x.

[ece372595-bib-0015] Eisentraut, M. 1928. Über die Baue und den Winterschlaf des Hamsters ( *Cricetus cricetus* L.). Vol. 3, 172–208. Zeitschrift für Säugetierkunde.

[ece372595-bib-0016] Esther, A. , C. Imholt , J. Perner , J. Schumacher , and J. Jacob . 2014. “Correlations Between Weather Conditions and Common Vole ( *Microtus arvalis* ) Densities Identified by Regression Tree Analysis.” Basic and Applied Ecology 15: 75–84. 10.1016/j.baae.2013.11.003.

[ece372595-bib-0014] European Environment Agency . n.d. “*Cricetus cricetus* (2013–2018). EU Biogeographical and Member States' Assessments [Article 17 Web Tool].” https://nature‐art17.eionet.europa.eu/article17/species/summary/?period=5&group=Mammals&subject=Cricetus+cricetus&region=.

[ece372595-bib-0017] Feoktistova, N. , I. G. Meschersky , P. L. Bogomolov , et al. 2020. “An Unintentional Experiment: Settlement of a Sinurbic Species, the Common Hamster ( *Cricetus cricetus* L., 1758), in a Newly Established City Park.” Biological Bulletin 47: 216–223. 10.1134/S1062359020020028.

[ece372595-bib-0018] Fischer, C. , and B. Schröder . 2014. “Predicting Spatial and Temporal Habitat Use of Rodents in a Highly Intensive Agricultural Area.” Agriculture, Ecosystems and Environment 189: 145–153. 10.1016/j.agee.2014.03.039.

[ece372595-bib-0019] Fischer, C. , and C. Wagner . 2016. “Can Agri‐Environmental Schemes Enhance Non‐Target Species? Effects of Sown Wildflower Fields on the Common Hamster ( *Cricetus cricetus* ) at Local and Landscape Scales.” Biological Conservation 194: 168–175. 10.1016/j.biocon.2015.12.021.

[ece372595-bib-0020] Geiger, F. , J. Bengtsson , F. Berendse , et al. 2010. “Persistent Negative Effects of Pesticides on Biodiversity and Biological Control Potential on European Farmland.” Basic and Applied Ecology 11: 97–105. 10.1016/j.baae.2009.12.001.

[ece372595-bib-0021] Gentili, S. , M. Sigura , and L. Bonesi . 2014. “Decreased Small Mammals Species Diversity and Increased Population Abundance Along a Gradient of Agricultural Intensification.” Hystrix 25: 39–44. 10.4404/hystrix-25.1-9246.

[ece372595-bib-0022] Grulich, I. 1981. “Die Baue des Hamsters ( *Cricetus cricetus* , Rodentia, Mammalia).” Folia Zoologica 30: 99–116.

[ece372595-bib-0023] Hacklander, K. , and S. Schai‐Braun . 2019. “*Lepus europaeus*.” The IUCN Red List of Threatened Species: e.T41280A45187424. 10.2305/IUCN.UK.2019-1.RLTS.T41280A45187424.en.

[ece372595-bib-0024] Hędrzak, M. J. , E. Badach , and S. A. Kornaś . 2021. “Preliminary Assumptions for Identification of the Common Hamster ( *Cricetus cricetus* ) as a Service Provider in the Agricultural Ecosystem.” Sustainability 13: 6793. 10.3390/su13126793.

[ece372595-bib-0025] Heroldová, M. , J. Šipoš , J. Suchomel , and J. Zejda . 2021. “Influence of Crop Type on Common Vole Abundance in Central European Agroecosystems.” Agriculture, Ecosystems and Environment 315: 107443. 10.1016/j.agee.2021.107443.

[ece372595-bib-0026] Hesselbarth, M. , M. Sciaini , K. A. With , K. Wiegand , and J. Nowosad . 2019. “Landscapemetrics: An Open‐Source R Tool to Calculate Landscape Metrics. R Package Version 2.1.1.” Ecography 42: 1648–1657. 10.1111/ecog.04617.

[ece372595-bib-0027] Huang, S. , L. Tang , J. P. Hupy , Y. Wang , and G. Shao . 2021. “A Commentary Review on the Use of Normalized Difference Vegetation Index (NDVI) in the Era of Popular Remote Sensing.” Journal of Forest Research 32: 1–6. 10.1007/s11676-020-01155-1.

[ece372595-bib-0028] Jacob, J. , P. Manson , R. Barfknecht , and T. Fredricks . 2014. “Common Vole ( *Microtus arvalis* ) Ecology and Management: Implications for Risk Assessment of Plant Protection Products.” Pest Management Science 70: 869–878. 10.1002/ps.3695. 24293354

[ece372595-bib-0029] Janova, E. , M. Heroldova , A. Konecny , and J. Bryja . 2011. “Traditional and Diversified Crops in South Moravia (Czech Republic): Habitat Preferences of Common Vole and Mice Species.” Mammalian Biology 76: 570–576. 10.1016/j.mambio.2011.04.003.

[ece372595-bib-0030] Katzman, E. A. , A. S. Sayan , P. L. Bogomolov , and A. B. Roumyantzev . 2023. “Influence of Landscape Transformation and the Anxiety Factor on the Distribution of Burrows of the Common Hamster ( *Cricetus cricetus* L., 1758) (Rodentia: Cricetidae) in the Conditions of the Park Zone of the City of Simferopol.” Biological Bulletin 50: 2630–2638. 10.1134/S1062359023100060.

[ece372595-bib-0031] Kayser, A. , and M. Stubbe . 2003. “Untersuchungen zum Einfluss unterschiedlicher Bewirtschaftung auf den Feldhamster *Cricetus cricetus* (L.), einer Leit‐ und Charakterart der Magdeburger Börde [On the Influence of Different Agricultural Management on the Common Hamster *Cricetus cricetus* (L.), a Key and Characteristic Species of the Landscape Magdeburger Börde].” Tiere Im Konflikt 7: 3–148.

[ece372595-bib-0032] Kayser, A. , U. Weinhold , and M. Stubbe . 2003. “Mortality Factors of the Common Hamster *Cricetus cricetus* at Two Sites in Germany.” Acta Theriologica 48: 47–57. 10.1007/BF03194265.

[ece372595-bib-0033] Kleijn, D. , F. Kohler , A. Báldi , et al. 2009. “On the Relationship Between Farmland Biodiversity and Land‐Use Intensity in Europe.” Proceedings of the Royal Society B 276: 903–909. 10.1098/rspb.2008.1509. 19019785 PMC2664376

[ece372595-bib-0034] Kletty, F. , M. L. Tissier , C. Kourkgy , et al. 2019. “A Focus on the European Hamster to Illustrate How to Monitor Endangered Species.” Integrative Zoology 14: 65–74. 10.1111/1749-4877.12375. 30585402

[ece372595-bib-0035] Kourkgy, C. , S. Marchandeau , G. Souchay , and J. Eidenschenck . 2019. “Evaluation of Innovative Agricultural Practices for Common Hamsters: Results of 5 Years of Survey in the Fields: The Final Results of the LIFE Alister Program.” 26th Meeting of the International Hamster Workgroup, 2019, Kerkrade, the Netherlands.

[ece372595-bib-0036] Kupfernagel, C. 2007. “Populationsdynamik und Habitatnutzung des Feldhamsters ( *Cricetus cricetus* ) in Südost‐Niedersachsen: Ökologie, Umsiedlung und Schutz.” Dissertation, Technische Universität Carolo‐Wilhelmina, Braunschweig, Germany.

[ece372595-bib-0037] La Haye, M. J. J. 2007. “The Effects of Hamster‐Friendly Management on the Hibernation Success of the Common Hamster.” Proceedings of the 15th Meeting of the lnternational Hamster Workgroup, Kerkrade, the Netherlands.

[ece372595-bib-0038] La Haye, M. J. J. , G. J. D. M. Müskens , R. J. M. van Kats , A. T. Kuiters , and H. Siepel . 2010. “Agri‐Environmental Schemes for the Common Hamster ( *Cricetus cricetus* ). Why Is the Dutch Project Successful?” Aspects of Applied Biology 100: 117–124.

[ece372595-bib-0039] La Haye, M. J. J. , K. Swinnen , A. T. Kuiters , H. Leirs , and H. Siepel . 2014. “Modelling Population Dynamics of the Common Hamster ( *Cricetus cricetus* ): Timing of Harvest as a Critical Aspect in the Conservation of a Highly Endangered Rodent.” Biological Conservation 180: 53–61. 10.1016/j.biocon.2014.09.035.

[ece372595-bib-0040] Landesanstalt für Landwirtschaft und Gartenbau Sachsen‐Anhalt . 2024a. Hinweise zum Intergrierten Pflanzenschutz: Feldmausbekämpfung—Umsetzung von Anwendungsbestimmungen beim Einsatz von Rodentiziden in Sachsen‐Anhalt. Pflanzenschutz‐Warndienst Allgemein.

[ece372595-bib-0041] Landesanstalt für Landwirtschaft und Gartenbau Sachsen‐Anhalt . 2024b. “Klimatische Wasserbilanz und Niederschlagsmenge in Sachsen‐Anhalt im hydrologischen Jahr 2023/2024 (November 2023 bis Oktober 2024)—Vergleich mit dem langjährigen Mittel 1991 bis 2020.” https://llg.sachsen‐anhalt.de/themen/agraroekologie‐und‐umwelt/agrarmeteorologie/klimatische‐wasserbilanz.

[ece372595-bib-0042] Li, H. , and J. Wu . 2004. “Use and Misuse of Landscape Indices.” Landscape Ecology 19: 389–399. 10.1023/B:LAND.0000030441.15628.d6.

[ece372595-bib-0043] Łopucki, R. , J. Wójciak , and D. Klich . 2024. “Climate Disturbances During Critical Periods Pose Risks to European Hamster Conservation Efforts.” Diversity and Distributions 30: e13899. 10.1111/ddi.13899.

[ece372595-bib-0044] Mammen, K. 2005. “Schutz Und Nutzung Des Feldhamsters in der Europäischen Union.” Beiträge zur Jagd‐ und Wildforschung 30: 401–407.

[ece372595-bib-0045] Marston, C. , F. Raoul , C. Rowland , et al. 2023. “Mapping Small Mammal Optimal Habitats Using Satellite‐Derived Proxy Variables and Species Distribution Models.” PLoS One 18: e0289209. 10.1371/journal.pone.0289209. 37590218 PMC10434852

[ece372595-bib-0046] Meinig, H. , P. Boye , M. Dähne , R. Hutterer , and J. Lang . 2020. Rote Liste und Gesamtartenliste der Säugetiere (Mammalia) Deutschlands. Naturschutz und Biologische Vielfalt, 170. 10.19213/972172.

[ece372595-bib-0047] Ministre de l'agriculture et de la souveraineté alimentaire . 2024. “Décret n° 2024–589 du 24 juin 2024 portant création d'un dispositif d'aide pour la préservation du hamster commun (*Cricetus cricetus*) dans les départements du Bas‐Rhin et du Haut‐Rhin.”

[ece372595-bib-0048] Olivier, T. , E. Thébault , M. Elias , B. Fontaine , and C. Fontaine . 2020. “Urbanization and Agricultural Intensification Destabilize Animal Communities Differently Than Diversity Loss.” Nature Communications 11: 2686. 10.1038/s41467-020-16240-6. PMC7264125 32483158

[ece372595-bib-0049] Out, M. E. , R. J. M. van Kats , A. T. Kuiters , G. J. D. M. Müskens , and M. J. J. La Haye . 2011. “Hard to Stay Under Cover: Seven Years of Crop Management Aiming to Preserve the Common Hamster ( *Cricetus cricetus* ) in the Netherlands.” Säugetierkundliche Informationen 8: 37–49.

[ece372595-bib-0050] Pinheiro, J. C. , D. M. Bates , and R Core Team . 2023. “nlme: Linear and Nonlinear Mixed Effects. R Package Version 3.1‐164.” 10.32614/CRAN.package.nlme.

[ece372595-bib-0051] Quemada, M. , and C. S. T. Daughtry . 2016. “Spectral Indices to Improve Crop Residue Cover Estimation Under Varying Moisture Conditions.” Remote Sensing 8: 660. 10.3390/rs8080660.

[ece372595-bib-0052] R Core Team . 2024. R: A Language and Environment for Statistical Computing. R Foundation for Statistical Computing. https://www.R‐project.org/.

[ece372595-bib-0053] Ridding, L. E. , S. C. L. Watson , A. C. Newton , C. S. Rowland , and J. M. Bullock . 2020. “Ongoing, but Slowing, Habitat Loss in a Rural Landscape Over 85 Years.” Landscape Ecology 35: 257–273. 10.1007/s10980-019-00944-2.

[ece372595-bib-0054] Ruscoe, W. A. , P. R. Brown , S. Henry , et al. 2023. “Effects of Harvesting and Stubble Management on Abundance of Pest Rodents ( *Mus musculus* ) in a Conservation Agriculture System.” Pest Management Science 79: 4757–4764. 10.1002/ps.7670. 37454375

[ece372595-bib-0055] Singh, M. , T. Tokola , Z. Hou , and C. Notarnicola . 2017. “Remote Sensing‐Based Landscape Indicators for the Evaluation of Threatened‐Bird Habitats in a Tropical Forest.” Ecology and Evolution 7: 4552–4567. 10.1002/ece3.2970. 28690786 PMC5496523

[ece372595-bib-0056] Stoate, C. , N. D. Boatman , R. J. Borralho , C. R. Carvalho , G. R. de Snoo , and P. Eden . 2001. “Ecological Impacts of Arable Intensification in Europe.” Journal of Environmental Management 63: 337–365. 10.1006/jema.2001.0473. 11826719

[ece372595-bib-0057] Surov, A. V. , A. Banaszek , P. L. Bogomolov , N. Feoktistova , and S. Monecke . 2016. “Dramatic Global Decrease in the Range and Reproduction Rate of the European Hamster *Cricetus cricetus* .” Endangered Species Research 31: 119–145. 10.3354/esr00749.

[ece372595-bib-0058] Thürkow, F. , M. Mohri , J. Ramstetter , and P. Alb . 2025. “LiDAR‐Based Detection of Field Hamster ( *Cricetus cricetus* ) Burrows in Agricultural Fields.” Sustainability 17: 6366. 10.3390/su17146366.

[ece372595-bib-0059] Tissier, M. L. , Y. Handrich , O. Dallongeville , J.‐P. Robin , and C. Habold . 2017. “Diets Derived From Maize Monoculture Cause Maternal Infanticides in the Endangered European Hamster Due to a Vitamin B3 Deficiency.” Proceedings of the Royal Society B 284: 20162168. 10.1098/rspb.2016.2168. 28100816 PMC5310035

[ece372595-bib-0060] Tissier, M. L. , Y. Handrich , J.‐P. Robin , et al. 2016. “How Maize Monoculture and Increasing Winter Rainfall Have Brought the Hibernating European Hamster to the Verge of Extinction.” Scientific Reports 6: 25531. 10.1038/srep25531. 27150008 PMC4858668

[ece372595-bib-0061] Tissier, M. L. , F. Kletty , J.‐P. Robin , and C. Habold . 2021. “Sustainable Agriculture: Nutritional Benefits of Wheat–Soybean and Maize–Sunflower Associations for Hibernation and Reproduction of Endangered Common Hamsters.” Sustainability 13: 13521. 10.3390/su132413521.

[ece372595-bib-0062] Tissier, M. L. , S. Marchandeau , C. Habold , Y. Handrich , J. Eidenschenck , and C. Kourkgy . 2019. “Weeds as a Predominant Food Source: A Review of the Diet of Common Hamsters *Cricetus cricetus* in Farmlands and Urban Habitats.” Mammal Review 49: 152–170. 10.1111/mam.12149.

[ece372595-bib-0063] Tkadlec, E. , M. Heroldová , V. Víšková , M. Bednář , and J. Zejda . 2012. “Distribution of the Common Hamster in the Czech Republic After 2000: Retreating to Optimum Lowland Habitats.” Folia Zoologica 61: 246–253. 10.25225/fozo.v61.i3.a9.2012.

[ece372595-bib-0064] van Wijk, R. , M. J. J. La Haye , R. J. M. van Kats , and G. J. D. M. Müskens . 2011. “Movement Characteristics of the Common Hamster ( *Cricetus cricetus* ) in Limburg, the Netherlands.” Säugetierkundliche Informationen 8: 79–91.

[ece372595-bib-0065] Villemey, A. , A. Besnard , J. Grandadam , and J. Eidenschenck . 2013. “Testing Restocking Methods for an Endangered Species: Effects of Predator Exclusion and Vegetation Cover on Common Hamster ( *Cricetus cricetus* ) Survival and Reproduction.” Biological Conservation 158: 147–154. 10.1016/j.biocon.2012.08.007.

[ece372595-bib-0066] Weidling, A. , and M. Stubbe . 1998a. “Feldhamstervorkommen in Abhängigkeit vom Boden.” Naturschutz und Landschaftspflege in Brandenburg 1: 18–21.

[ece372595-bib-0067] Weidling, A. , and M. Stubbe . 1998b. “Eine Standardmethode zur Feinkartierung von Feldhamsterbauen.” In Ökologie Und Schutz Des Feldhamsters, 259–276. Halle/Saale.

[ece372595-bib-0068] Weinhold, U. 2009. “Draft European Action Plan for the Conservation of the Common Hamster (*Cricetus cricetus*, L. 1758).” 3rd Version. Prelimenary Document. Council of Europe Document T‐PVS/Inf(2008)9.

[ece372595-bib-0069] Weinhold, U. , and A. Kayser . 2006. Der Feldhamster: *Cricetus cricetus* . 1st ed. Die neue Brehm‐Bücherei, 625. Westarp Wissenschaften. Hohenwarsleben.

[ece372595-bib-0070] Yue, J. , and Q. Tian . 2020. “Estimating Fractional Cover of Crop, Crop Residue, and Soil in Cropland Using Broadband Remote Sensing Data and Machine Learning.” International Journal of Applied Earth Observation and Geoinformation 89: 102089. 10.1016/j.jag.2020.102089.

