# Supplementary material for: Unimpressed by the Environment?—Local and Landscape Scale Effects on the Common Hamster in a Simple Agricultural Landscape
Source: Ecol Evol. 2025 Dec 22;15(12):e72595. doi: 10.1002/ece3.72595 (PMC12720141; doi:10.1002/ece3.72595)
Supplement: Supplementary file 2 — Appendix S1: ece372595‐sup‐0002‐AppendixS1.docx. [file ECE3-15-e72595-s001.docx]

Supplementary Information for
 Unimpressed by the environment? - Local and landscape scale effects on the common hamster in a simple agricultural landscape

Animal Conservation

Pia Stein, Saskia Jerosch, Marion Pause, Christina Fischer


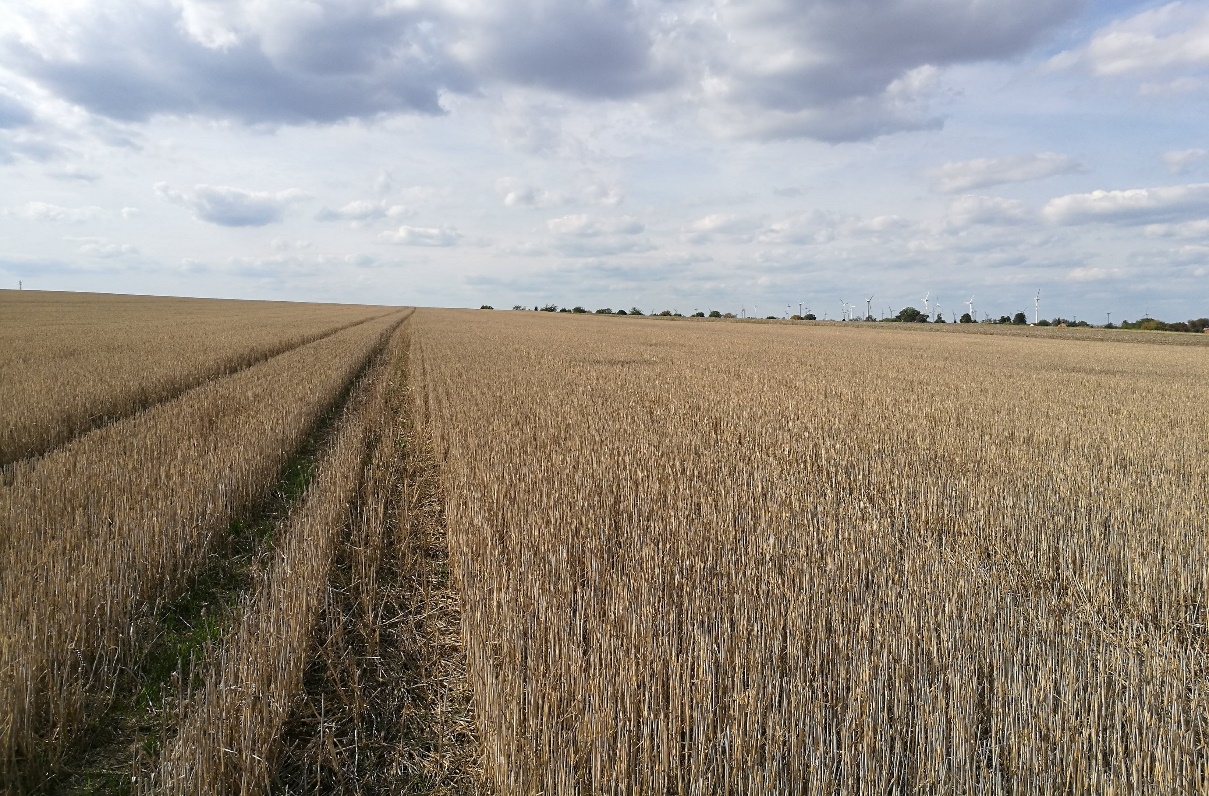
Figure A.1: Field with the hamster protection measure high cut harvest, Photo by Pia Stein, 2022

Table A.1. Correlations of landscape composition and configuration variables measured within a radius of 500 m around a field centre and Euclidean distances from field edges to the nearest forest and urban fabric. Spearman's rank correlation coefficients (r_s_) are given and values highlighted in grey indicate significant correlations (r_s_ > 0.7, Dormann et al., 2013) (*n* = 19).

|  | Winter cereals | Oil seeds | Uncultivated cover | Crop diversity | Mean field size | Edge density | Distance nearest Forest | Distance nearest urban fabric |
| --- | --- | --- | --- | --- | --- | --- | --- | --- |
| Winter cereals | - |  |  |  |  |  |  |  |
| Oil seeds | 0,12 | - |  |  |  |  |  |  |
| Uncultivated cover | -0,31 | -0,42 | - |  |  |  |  |  |
| Crop diversity | -0.1 | 0.47 | 0.22 | - |  |  |  |  |
| Mean field size | 0.37 | -0.07 | -0.02 | -023 | - |  |  |  |
| Egde density | -0.06 | 0.06 | -0.23 | 0.42 | -0.58 | - |  |  |
| Distance nearest forest | 0.45 | 0.12 | 0.17 | 0.08 | 0.51 | -0.37 | - |  |
| Distance nearest urban fabric | 0.75 | 0.11 | 0.06 | 0.17 | 0.11 | -0.08 | 0.22 | - |

Table A.2 Selected best models with the parameters NDVI and NDTI with an evidence ratio ΔAICc ≤ 2 are shown. The table shows Akaike’s information criterion for small sample size corrections (AICc), ΔAICc values, NDVI = Normalized difference vegetation index, NDTI = Normalized difference tillage index, DF = degree of freedom, w = Akaike Weights

| Model | Intercept | NDVI | NDTI | DF | AICc | ΔAICc | w |
| --- | --- | --- | --- | --- | --- | --- | --- |
| 1 | -0.31 | - | - | 1 | 28.1 | 0 | 0.34 |
| 2 | -1.71 | 7.33 | - | 2 | 28.37 | 0.27 | 0.3 |
| 3 | -1.82 | - | 16.83 | 2 | 28.48 | 0.38 | 0.28 |

Table A.3 Selected best models of landscape composition within 500 m of field center with evidence ratio ΔAICc ≤ 3. Akaike’s information criterion for small sample size corrections (AICc), ΔAICc values, DF = degree of freedom, w = Akaike Weights

| Model | Intercept | Winter cereals | Oil seeds | Uncultivated land | Crop diversity | DF | AICc | ΔAICc | w |
| --- | --- | --- | --- | --- | --- | --- | --- | --- | --- |
| 1 | -5.11 | 0.1 | - | - | - | 2 | 23.03 | 0 | 0.39 |
| 2 | -0.59 | 0.11 | - | - | -3.12 | 3 | 24.53 | 1.5 | 0.19 |
| 3 | -6.07 | 0.11 | - | 0.02 | - | 3 | 25.51 | 2.48 | 0.11 |
| 4 | -4.96 | 0.1 | -0.03 | - | - | 3 | 25.68 | 2.65 | 0.1 |

Table A.4 Selected best models of landscape configuration within 500 m of field center and the closest distance to forests with evidence ratio ΔAICc ≤ 3. Akaike’s information criterion for small sample size corrections (AICc), ΔAICc values, DF = degree of freedom, w = Akaike Weights

| Model | Intercept | Edge Density | Mean field size | Distance to the nearest forest | DF | AICc | ΔAICc | w |
| --- | --- | --- | --- | --- | --- | --- | --- | --- |
| 1 | 3.22 | -0.03 | - | - | 2 | 27.31 | 0 | 0.32 |
| 2 | -0,32 | - | - | - | 1 | 28.1 | 0.79 | 0.21 |
| 3 | -1.72 | - | 0.33 | - | 2 | 28.53 | 1.21 | 0.17 |
| 4 | 1.92 | -0.02 | 0.15 | - | 3 | 29.83 | 2.52 | 0.09 |
| 5 | 3.41 | -0.03 | - | -0.00 | 3 | 30.15 | 2.83 | 0.08 |

References

Dormann, C. F., Elith, J., Bacher, S., Buchmann, C., Carl, G., Carré, G., Marquéz, J. R. G., Gruber, B., Lafourcade, B., Leitão, P. J., Münkemüller, T., McClean, C., Osborne, P. E., Reineking, B., Schröder, B., Skidmore, A. K., Zurell, D., & Lautenbach, S. (2013). Collinearity: a review of methods to deal with it and a simulation study evaluating their performance. Ecography, 36, 27–46. https://doi.org/10.1111/j.1600-0587.2012.07348.x
